# Triple‑negative breast cancer cell‑derived piR‑31115 promotes the proliferation and migration of endothelial cells via METTL3‑mediated m6A modification of YAP1

**DOI:** 10.3892/or.2025.8867

**Published:** 2025-01-16

**Authors:** Shan-Mei Du, Na Li, Wen-Jing Xu, Kui Liu

**Affiliations:** 1 School of Medicine, Zibo Vocational Institute, Zibo, Shandong 255300, P.R. China; 2 Department of Gastroenterology, Zhongda Hospital, Affiliated Hospital of Southeast University, Nanjing, Jiangsu 210009, P.R. China; 3 Department of Hepatobiliary and Pancreatic Surgery, The Second Affiliated Hospital, Zhejiang University School of Medicine, Hangzhou, Zhejiang 310009, P.R. China; 4 Center of Translational Medicine, Zibo Central Hospital, Zibo, Shandong 255036, P.R. China

**Keywords:** triple-negative breast cancer, Piwi-interacting RNA-31115, HMEC-1, METTL3, Yes-associated protein 1, IGFBP2

## Abstract

Triple-negative breast cancer (TNBC), a highly malignant breast cancer subtype with a pronounced metastatic propensity, forms the focus of the present investigation. MDA-MB-231, a prevalently utilized TNBC cell line in cancer research, was employed. In accordance with the tumour angiogenesis theory, cancer cells are capable of instigating angiogenesis and the formation of a novel vascular system within the tumour microenvironment, which subsequently sustains malignant proliferation and metastasis. Consequently, impeding the growth of tumour blood vessels holds substantial significance in suppressing TNBC metastasis. Piwi-interacting RNAs (piRNAs), a category of endogenous non-coding RNAs, have been demonstrated to modulate cancer progression. However, studies regarding the role of piRNAs in regulating angiogenesis within cancer cells are relatively scant. In the present study, via cell co-culture experiments, it was revealed that piR-31115 (a kind of piRNA) in MDA-MB-231 cells notably enhanced the recruitment of a human microvascular endothelial cell line (HMEC-1). Moreover, the conditioned medium (CM, which was obtained from MDA-MB-231 cells via a specific culturing methodology and was employed for the subsequent treatment of HMEC-1 cells to explore its impacts on the biological behaviors such as the proliferation and migration of HMEC-1 cells) derived from MDA-MB-231 cells with upregulated piR-31115 expression stimulated the proliferation and migration of HMEC-1 cells. These findings suggest that piR-31115 in MDA-MB-231 cells may play a pivotal role in modulating tumour angiogenesis. Further studies disclosed that the CM from MDA-MB-231 cells augmented the N6-methyladenosine (m6A) RNA modification level via METTL3 in HMEC-1 cells. Transcriptome sequencing revealed that METTL3 functions as an m6A writer protein for Yes-associated protein 1 (YAP1), which exerts a positive influence on promoting the proliferation and migration of HMEC-1 cells. Concurrently, the IGF2BP plays a crucial role in stabilizing YAP1 protein expression. Collectively, the present findings identified a signalling pathway through which MDA-MB-231 cells induce HMEC-1 cell proliferation and migration by regulating m6A RNA methylation.

## Introduction

Triple-negative breast cancer (TNBC) represents the most aggressive subtype among all breast cancers, accounting for ~15% of breast cancer cases (1). With respect to the fact that TNBC does not express the estrogen receptor, progesterone receptor, or human epidermal growth factor receptor-2 (HER-2), patients with TNBC cannot obtain benefits from endocrine- or HER-2-targeted therapies. The treatment protocols for TNBC encompass radiotherapy, chemotherapy and surgery procedures. Presently, there have been some advancements in the immunotherapy of TNBC as well (2,3). Nevertheless, TNBC has a comparatively high vascular density. This feature enables cancer cells to multiply swiftly and metastasize to distant tissues (4,5), which in turn still results in the reduction of the progression-free survival (PFS) period among patients with metastatic TNBC (6,7). Consequently, elucidating the underlying molecular mechanisms of angiogenesis in TNBC holds great clinical significance.

Non-coding RNAs (ncRNAs) play crucial roles in the genetic evolution of organisms (8–10). Piwi-interacting RNAs (piRNAs), which belong to a type of ncRNAs, need to bind to PIWI family proteins to exert a variety of biological effects (11). In reproductive stem cells, piRNA/PIWI complexes maintain the integrity of the transposon genome by silencing transposons (12). Besides the mammalian reproductive system, piRNAs can be expressed in numerous tissues within the human body (13–15). Previously, it was reported that piRNAs are associated with cancer. For instance, piR-823 can suppress the proliferation of gastric cancer cells (16). The expression of piR-823 is elevated in multiple myeloma cells, and it can subsequently promote their proliferation by influencing apoptosis (17). piR-651 can impede the apoptosis of lung cancer cells (18). In colon cancer, the downregulation of piR-1245 expression leads cells to undergo apoptosis (19). These findings suggest that piRNAs have a regulatory function in the development of cancer.

A recent study revealed that piR-31115 is aberrantly elevated in clear cell renal cancer and it was established that the effect of piR-31115 can enhance cancer cell invasion (20). Koduru *et al* (21) reported that piR-hsa-1254 (also known as piR-31115) is upregulated in TNBC tissue samples. However, the role of piR-31115 in TNBC has not yet been reported. In the present study, it was discovered that piR-31115 derived from MDA-MB-231 cells promotes the proliferation and migration of HMEC-1 cells by modulating N6-methyladenosine (m6A) modification. The results offer a perspective for further exploration of angiogenesis induced by TNBC.

## Materials and methods

### Tissue sample collection and preparation

TNBC tissue specimens were obtained for the present study. A total of 27 female patients, with ages ranging from 38 to 57 years old, were treated at the Department of Breast and Thyroid Surgery in Zibo Central Hospital between January 2022 and December 2023, provided the TNBC and adjacent normal tissue samples. Both the tumour tissues and the corresponding adjacent normal tissues were histologically confirmed. Immediately after the surgical procedure, the tissue specimens were placed into cryovials, snap-frozen, and then stored in liquid nitrogen until further use. Every participant signed a statement of informed consent. The protocol for the utilization of patient samples was approved (approval no. 202102005) by the Ethics Committee of Zibo Central Hospital (Zibo, China).

### Cell culture

HMEC-1 cells were kindly provided by Dr Z. Wang (Zhongda Hospital, Affiliated Hospital of Southeast University, Nanjing, China). Normal human breast epithelial cells (MCF-10A) were a gift from Dr XC. Sun (Jiangsu University, Zhenjing, China). MDA-MB-231 cells were provided by Dr H. Yang (Tai'an City Central Hospital, Affiliated Hospital of Qingdao University, Tai'an, China). HMEC-1 cells were cultured in DMEM/F-12 (cat. no. SH30023.01; HyClone; Cytiva) supplemented with 10% (v/v) fetal bovine serum (FBS; cat. no. 11012-8611; Every Green; http://www.hzsjq.com/). MDA-MB-231 cells were maintained in Leibovitz's L-15 medium (cat. no. CM10045; MacGene; http://www.macgene.com/) supplemented with 10% (v/v) FBS (cat. no. 11012-8611; Every Green). MCF-10A cells were maintained in mammary epithelial cell medium supplemented with 5% horse serum and 1% growth medium (cat. no. ZQ-1311; Zhongqiao Xinzhou Biotechnology Co., Ltd.). All the cells were incubated in a thermostatic incubator at 37°C with 5% CO_2_ (for HMEC-1 and MCF-10A) or without CO_2_ (for MDA-MB-231).

### RNA extraction and reverse transcription-quantitative PCR (RT-qPCR)

The Beyozol (cat. no. R0011; Beyotime Institute of Biotechnology) was utilized to lyse the tissues and cells. The total RNA was extracted using a kit (cat. no. G3607-50T; Wuhan Servicebio Technology Co., Ltd.). cDNA was synthesized from the total RNA by means of a kit (cat. no. TSK302S; Tsingke Biological Technology) according to the manufacturer's instructions. The cDNA levels were detected with an Applied Biosystems^®^ 7500 (Thermo Fisher Scientific, Inc.) using a kit (cat. no. D7260; Beyotime Institute of Biotechnology). The thermocycling conditions for qPCR were as follows: Initial denaturation at 95°C for 10 min, followed by 40 cycles of denaturation at 95°C for 15 sec, annealing at 60°C for 30 sec, and extension at 72°C for 30 sec. The comparative cycle threshold (Ct) value method was employed to determine the fold-differences in expression levels in relation to those in U6 snRNA or β-actin (22). The sequences of primers used are presented in Table I.

### Plasmids and small interfering RNA (siRNA) transfection

Plasmids harboring METTL3, HA-Ub, Myc-YAP1 and siRNAs targeting YAP1 (si-YAP1), METTL3 (si-METTL3), IGF2BP1 (si-IGF2BP1), IGF2BP2 (si-IGF2BP2), IGF2BP3 (si-IGF2BP3) and si-NC were synthesized by Tsingke Biological Technology. The concentration of nucleic acid used was 50 nM. These were transfected into cells using TSnanofect V1 (cat. no. TSV404; Tsingke Biological Technology) at a temperature of 37°C for a duration of 6 h. The time interval between transfection and subsequent experimentation was 48 h. All the siRNA sequences are presented in Table II.

### Lentiviral transduction

The lentiviral plasmids for overexpressing piR-31115 (LV-piR-31115), knocking down piR-31115 (LV-piR-31115 inhibitor) and the negative control (LV-NC and LV-inhibitor NC) were procured from Shanghai GenePharma Co., Ltd. (cat. no. LV2022-7704). The lentiviral transduction was carried out using the 2nd generation system. The interim cell line employed in this process was 293T, which was provided by Shanghai GenePharma Co., Ltd. For transfection, the quantity of the lentiviral plasmid used was 4 µg per 6-well plate, and the ratio of the lentivirus, packaging and envelope plasmids was optimized as transfer plasmid: psPAX2: pMD2.G=4:3:1. The transfection procedure was performed at a temperature of 37°C, and it lasted for 6 h. After 48 h, the cell supernatant was collected and the lentiviral particles were then collected through ultracentrifugation. To infect the target cells, a multiplicity of infection of 50 was applied. The transduction of the cells of interest lasted ~6 h. Notably, there was a time interval of 48 h between the completion of transduction and the subsequent experimentation. Stably transduced MDA-MB-231 cell lines (MDA-MB-231-LV-NC, MDA-MB-231-LV-piR-31115, MDA-MB-231-LV-inhibitor NC, and MDA-MB-231-LV-piR-31115 inhibitor) were obtained via through selection with puromycin at a concentration of 1 µg/ml (cat. no. ST551; Beyotime Institute of Biotechnology).

### Cell co-culture assay

An equal number of stably transduced MDA-MB-231 cells were seeded in 24-well plates. The wells without cells served as the control group. Initially, the lower chamber was filled with Leibovitz's L-15 medium when MDA-MB-231 cells were seeded. Before the cell co-culture, the Leibovitz's L-15 medium in the lower chamber was removed and replaced with DMEM/F-12 supplemented with 10% (v/v) fetal bovine serum. Then, a Transwell insert (cat. no. 3422; Corning, Inc.) with a pore size of 8 µm was placed. Subsequently, 1×10^4^ HMEC-1 cells were added to the upper chamber of the Transwell insert, where the medium used was DMEM/F-12 without fetal bovine serum, and cultured for 12 h at 37°C. The HEMC-1 cells that had migrated to the lower chamber were fixed with 4% paraformaldehyde at room temperature for 30 min and then stained with a 0.1% crystal violet solution at room temperature for 30 min. The number of migratory cells was counted in three randomly selected fields (at a low magnification of ×100) using a light microscope.

### Production of the conditioned medium (CM)

Firstly, an equal quantity of stably transduced MDA-MB-231 cells were meticulously inoculated into a culture dish and permitted to adhere to the surface for a duration of time. Subsequently, the original culture medium was gingerly removed, and a serum-free DMEM/F-12 mixture was introduced into the dish for culturing. After 24 h, the resultant CM, which now encompassed the secreted factors, was filtered through a 0.22-µm filter (cat. no. SLHV033RS; MilliporeSigma) to eliminate any cellular debris or large particles. The filtered CM was then apportioned and promptly frozen at −80°C until it was requisite for further experiments. This painstaking process of CM generation ensured the collection of a cell-CM that could be utilized to explore its effects on other cell types, such as HMEC-1 cells, in subsequent assays.

### Cell counting kit-8 (CCK-8) assay

First, a total of 2×10^3^ HMEC-1 cells (either transfected or non-transfected) per well were seeded in 96-well plates and allowed to attach. Subsequently, they were treated with CM for 24 h. Finally, 10 µl of CCK-8 reagent (cat. no. C0048S; Beyotime Institute of Biotechnology) was mixed with 90 µl of DMEM/F-12 cell medium and added to one well of the 96-well plate. The incubation with CCK-8 was performed at 37°C for 0.5 h, and the absorbance was measured at a wavelength of 450 nm.

### Transwell migration assays

Transwell inserts (cat. no. 3422; Corning, Inc.) were employed to conduct cell migration assays. First, HMEC-1 cells (either transfected or non-transfected) were treated with CM for 24 h. Then, 1×10^4^ cells were resuspended in 0.1 ml of serum-free DMEM/F-12 and seeded in the upper chamber. DMEM/F-12 containing 10% serum was added to the lower chamber. After culturing for 12 h at 37°C, the cells that had migrated to the lower chamber were fixed and stained with a crystal violet solution. The number of migratory cells was counted in three randomly selected fields (at a low magnification of ×100).

### Western blotting (WB)

RIPA lysis buffer (cat. no. P0013B; Beyotime Institute of Biotechnology) was used to extract the total cellular protein. The proteins were separated into cytoplasmic and nuclear fractions using a kit (cat. no. P0027; Beyotime Institute of Biotechnology). The protein determination was performed using the bicinchoninic acid (BCA) method. Proteins were separated by 10% SDS-PAGE (cat. no. P0012A; Beyotime Institute of Biotechnology) and then transferred to PVDF membranes (cat. nos. ISEQ00010 and IPVH00010; Merck KGaA). A protein mass of 100 micrograms was loaded per lane. The membranes were blocked with 5% defatted milk/TBST (containing 0.1% Tween) for 1 h at room temperature, and then incubated with primary antibodies against METTL3 (1:1,000; cat. no. GB114688; Wuhan Servicebio Technology Co., Ltd.), PIWIL4 (1:200; cat. no. sc-517215; Santa Cruz Biotechnology, Inc.), VEGFA (1:5,000; cat. no. 81323-2-RR), YAP1 (1:5,000; cat. no. 66900-1-lg), IGF2BP1 (1:10,000; cat. no. 22803-1-AP), IGF2BP2 (1:2,000; cat. no. 11601-1-AP), IGF2BP3 (1:10,000; cat. no. 14642-1-Ig), proliferating cell nuclear antigen (1:10,000; cat. no. 60097-1-Ig), HA (1:10,000; cat. no. 66006-2-Ig), Myc (1:5,000; cat. no. 66003-2-Ig; all from Proteintech Group, Inc.) and β-actin (1:1,000; cat. no. GB15003; Wuhan Servicebio Technology Co., Ltd.) overnight at 4°C. Next, the membranes were incubated with horseradish peroxidase-conjugated secondary antibodies (1:10,000; cat. nos. GB23301 and GB23303; Wuhan Servicebio Technology Co., Ltd.) at room temperature for 1 h. The pre-stained protein marker was purchased from Wuhan Servicebio Technology Co., Ltd. (cat. no. G2083). The protein bands were developed using an enhanced chemiluminescence reagent (cat. no. P0018; Beyotime Institute of Biotechnology).

### Coimmunoprecipitation (Co-IP) assays

Co-IP of the lysates was carried out using a kit (cat. no. P2179; Beyotime Institute of Biotechnology). The lysis buffer used was Lysis Buffer from the kit (containing components and concentrations as provided by the manufacturer). For each IP reaction, 500 µl of lysate was used. BeyoMag^™^ Protein A+G magnetic beads (20 µl per 500 µl sample) were used. Protein A+G can bind to the Fc end of the antibody specifically. After incubation for 30 min, the Protein A+G magnetic beads-antibody mixture (beads-Ab complex) was formed. Then the sample was added, and the sample could be specifically recognized by the Fab end of the antibody to form the Protein A+G magnetic beads-antibody-antigen immune complex (beads-Ab-Ag complex). The immunocomplex was washed to remove unbound proteins. Centrifugation steps included centrifugation at 12,000 × g at 4°C for 5 min during the sample preparation process. WB was performed to evaluate protein expression in the samples obtained through immunoprecipitation with anti-PIWIL4 antibodies (1:100; cat. no. sc-517215; Santa Cruz Biotechnology, Inc.). The input and IgG groups were utilized as the positive and negative controls, respectively.

### Total RNA m6A quantification

Total RNA m6A quantification was carried out using a kit (cat. no. P-9005; EpiGentek). The cells (either transfected or non-transfected) were treated with CM for 24 h. Subsequently, the RNA extracted from the cells with TRIzol reagent (Thermo Fisher Scientific, Inc.) was combined with the capture antibody. During multiple incubations steps, colorimetric measurement of the m6A content was performed at a wavelength of 450 nm.

### Methylated RNA immunoprecipitation (MeRIP)

MeRIP was carried out using a kit (cat. no. Bes5203-2; Guangzhou Bersinbio Co., Ltd.). Briefly, the RNA of cells was extracted with Beyozol reagent and fragmented by ultrasonication. The RNA was incubated with an anti-m6A antibody at 4°C for 4 h and then with magnetic beads. The enrichment of m6A-containing mRNAs was then analysed by RT-qPCR.

### Transcriptome sequencing and pathway enrichment analysis

The RNA from cells was extracted with Beyozol and then used to construct an RNA library using VAHTS Universal V6 RNA-seq Library Prep Kit (cat. no. NR604; Shanghai OE Biotech Co., Ltd.). The quality/integrity of the processed RNA samples was verified by Agilent 2100 Bioanalyzer. The RNA library was sequenced on the Illumina NovaSeqTM 6000 platform by OE Biotech, Inc. with a sequencing type of 150-bp paired end using VAHTS Universal V6 RNA-seq Library Prep Kit (cat. no. NR604; Shanghai OE Biotech Co., Ltd.). The loading concentration of the final library was 5 nM. The pathway enrichment analysis was performed using the Kyoto Encyclopedia of Genes and Genomes (KEGG) database (https://www.genome.jp/kegg/). This database was utilized to identify the potential signaling pathways related to the genes of interest. The analysis was based on the publicly available data and algorithms in the KEGG database to explore the biological functions and pathways associated with the differentially expressed genes.

### Statistical analysis

Statistical analyses were performed using the Prism software (version 5.0; Dotmatics). *In vitro* data (mean ± standard deviation) were obtained from three independent experiments. To evaluate the statistical significance of the differences between two separate groups, the following rules were applied: for tissue samples, Student's t-test (paired) was used, while for other samples, Student's t-test (unpaired) was applied. For comparisons among more than two groups, one-way analysis of variance (ANOVA) was utilized, followed by Newman-Keuls multiple comparison test. P<0.05 was considered to indicate a statistically significant difference.

## Results

### MDA-MB-231 cell-derived piR-31115 promotes the proliferation and migration of HMEC-1 cells

**Figure 1.**
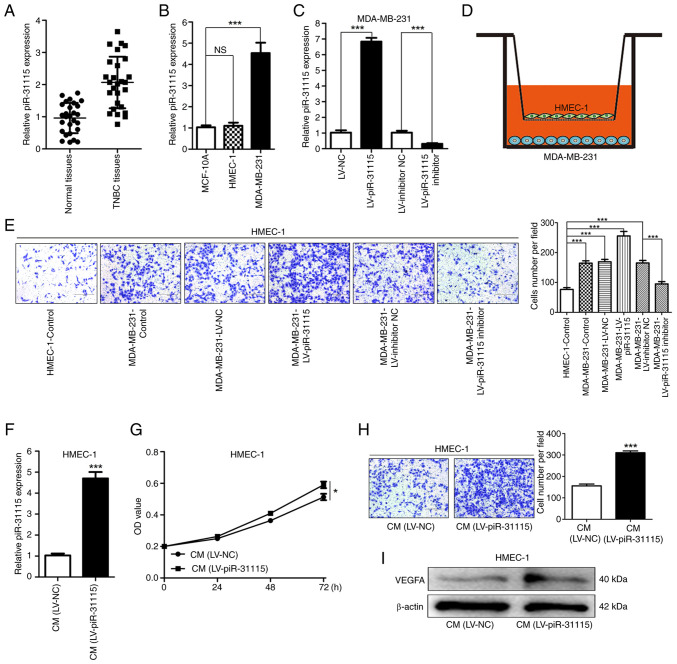
MDA-MB-231 cell-derived piR-31115 promotes HMEC-1 cell proliferation and migration. (A-C) RT-qPCR was used to examine piR-31115 expression in tissue specimens and cells. (D) A schematic shows the cell co-culture assay setup. (E) Transwell assays were used to assess HMEC-1 cell migration when co-cultured with MDA-MB-231 cells (magnification, ×100). (F) RT-qPCR was utilized to determine piR-31115 levels in HMEC-1 cells post CM treatment. (G) Cell Counting Kit-8 assay was used to evaluate cell proliferation after CM treatment. (H) Transwell assays assessing HMEC-1 cell migration after CM treatment (magnification, ×100). (I) VEGFA protein levels in CM-treated HMEC-1 cells were analysed by western blotting. Data are derived from three independent experiments. *P<0.05 and ***P<0.001. piR, Piwi-interacting RNA; RT-qPCR, reverse transcription-quantitative PCR; CM, conditioned medium; NC, negative control; LV, lentivirus; NS, not significant.

To investigate the potential role of piR-31115 in TNBC, RT-qPCR was conducted on breast cancer and adjacent normal tissues from 27 patients with TNBC to detect its expression. The results evidently indicated that piR-31115 was significantly more highly expressed in TNBC tissues compared with adjacent normal ones (Fig. 1A), preliminarily suggesting its potential involvement in TNBC development. Using MCF-10A cells as a control, the expression levels of piR-31115 were evaluated in MDA-MB-231 and HMEC-1 cells. The results demonstrated that the expression level of piR-31115 in MDA-MB-231 cells was higher than that in MCF-10A cells. Additionally, no significant difference was detected in piR-31115 expression between MCF-10A cells and HMEC-1 cells (Fig. 1B). Lentiviral vector transduction was carried out to obtain MDA-MB-231 cells in which piR-31115 was stably overexpressed or knocked down (Fig. 1C). Through cell co-culture experiments, it was discovered that MDA-MB-231 cells exhibited chemotactic effects on HMEC-1 cells. After the expression of piR-31115 in MDA-MB-231 cells was upregulated, its chemotactic effect on HEMC-1 cells was markedly enhanced. When the expression of piR-31115 in MDA-MB-231 cells was interfered with, its chemotactic effect on HMEC-1 cells was reduced (Fig. 1D and E). These findings suggested that MDA-MB-231 cell-derived piR-31115 can regulate the biological behaviour of HMEC-1 cells. HMEC-1 cells were treated with medium from MDA-MB-231 cells with overexpression of piR-31115. The RT-qPCR results showed that the piR-31115 expression level was increased in HMEC-1 cells (Fig. 1F). Further examination of HMEC-1 cells after CM treatment revealed that their proliferation and migration abilities were enhanced (Fig. 1G and H). WB analysis demonstrated that the VEGFA protein expression level increased in HMEC-1 cells after CM treatment (Fig. 1I). These data suggest that MDA-MB-231 cell-derived piR-31115 plays a crucial role in promoting angiogenesis.

### MDA-MB-231 cell-derived piR-31115 increases the m6A level in HMEC-1 cells via METTL3

**Figure 2.**
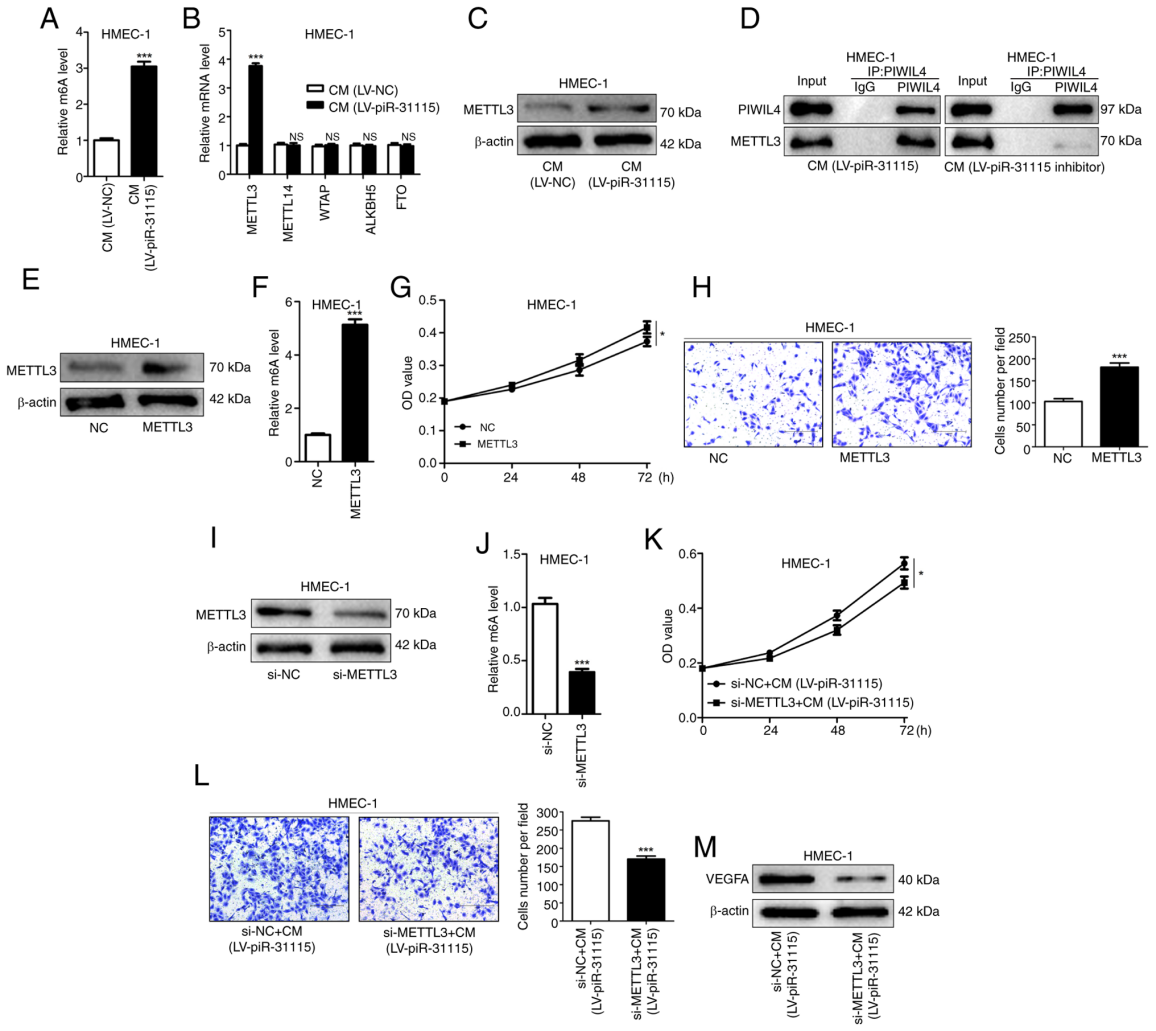
piR-31115 mediates m6A methylation via METTL3 regulation. (A) m6A RNA methylation quantification kit was used to detect m6A levels in CM-cultured HMEC-1 cells. (B) Reverse transcription-quantitative PCR ascertained METTL3, METTL14, WTAP, ALKBH5 and FTO mRNA levels. (C) METTL3 protein levels after CM treatment were analysed by WB. (D) Co-immunoprecipitation of endogenous PIWIL4 and METTL3 from CM-treated cell lysates, then analysed by WB. (E and F) Kit-detected m6A levels in METTL3-overexpressing cells. (G) CCK-8 assay was used to assess proliferation of METTL3-overexpressing cells. (H) Transwell assays evaluating migration of METTL3-overexpressing cells (magnification, ×100). (I and J) Kit-detected m6A levels in METTL3-knockdown cells. (K) CCK-8 assay gauged proliferation of METTL3-knockdown, CM-treated cells. (L) Transwell assays measured their migration. (M) WB was utilized to analyse VEGFA levels in METTL3-knockdown, CM-treated cells (magnification, ×100). Data are derived from three independent experiments. *P<0.05 and ***P<0.001. piR, Piwi-interacting RNA; m6A, N6-methyladenosine; CM, conditioned medium; WB, western blotting; CCK-8, Cell Counting Kit-8; NC, negative control; LV, lentivirus; NS, not significant; si-, small interfering.

In recent years, m6A methylation has been established to play a significant role in the metastasis of cancer (23,24). A substantial increase was detected in total methylation in HMEC-1 cells treated with CM (Fig. 2A). These findings indicate that piR-31115 derived from MDA-MB-231 cells can regulate m6A methylation levels in HMEC-1 cells. m6A methylation is a dynamic and reversible modification regulated by methyltransferases (METTL3, METTL14 and WTAP) and demethylases (ALKBH5 and FTO). The mRNA expression levels of METTL3, METTL14, WTAP, ALKBH5 and FTO were determined, and the results showed that only the expression of METTL3 increased in HMEC-1 cells under CM treatment (Fig. 2B). Further detection of METTL3 protein expression levels by WB demonstrated that METTL3 protein expression levels rose under CM treatment (Fig. 2C). piRNAs need to bind to the PIWI family of proteins to exert a variety of biological effects (11). Wang *et al* (25) have experimentally confirmed that the expression of PIWIL4 is increased in MDA-MB-231 and promotes its metastasis. Meanwhile, they also proposed the possibility that PIWIL4 cooperates with piRNA to achieve its functions (25). In the present study, co-IP experiments were utilized and it was found that PIWIL4 and METTL3 were bound to each other in HMEC-1 cells treated with CM. When HMEC-1 cells were treated with CM from MDA-MB-231 cells with piR-31115 knockdown, the binding effect of PIWIL4 and METTL3 was diminished (Fig. 2D). These results suggest that METTL3 is a target gene regulated by the piR-31115-PIWIL4 complex. The upregulation of METTL3 expression in HMEC-1 cells not only augmented the overall level of m6A modification but also significantly enhanced their proliferative and migratory capacities (Fig. 2E-H). After interfering with the expression of METTL3 in HMEC-1 cells, the stimulatory effect of CM on cell proliferation and migration was attenuated, concurrently leading to a downregulation of VEGFA expression (Fig. 2I-M). These results comprehensively indicate that METTL3 is an important regulatory gene of piR-31115 and that CM elevates its m6A modification level through the upregulation of METTL3 expression in HMEC-1 cells, thereby resulting in increased cell proliferation and migration, which is beneficial for the formation of microvessels.

### The influence of METTL3 on signaling pathways in HMEC-1 Cells treated with CM

**Figure 3.**
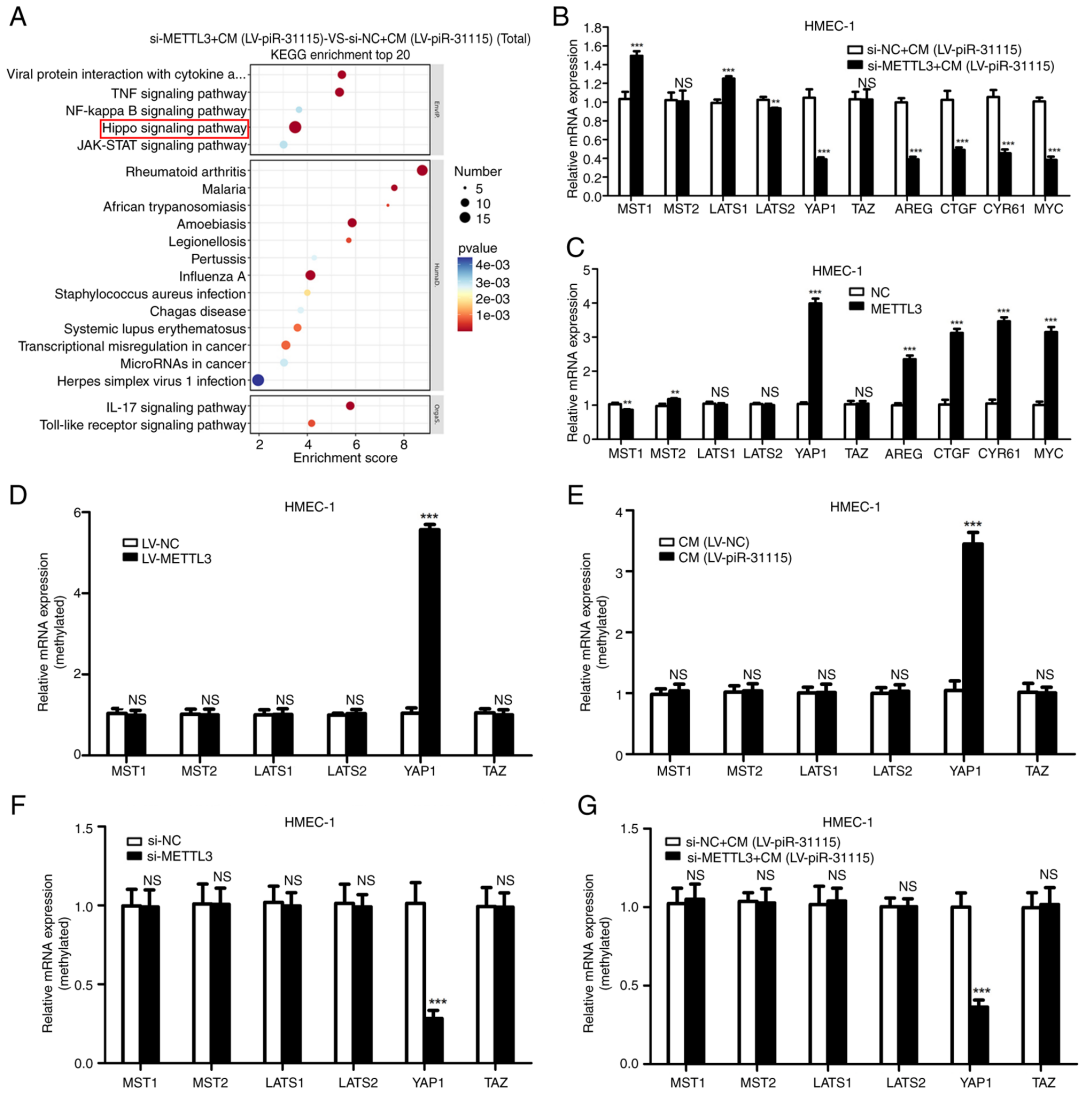
Identification of METTL3 target genes via RNA-sequencing. (A) KEGG pathway enrichment analysis on METTL3-knockdown, CM-treated HMEC-1 cells. (B and C) Reverse transcription-quantitative PCR was used to examine the Hippo signalling pathway-related genes (MST1, MST2, LATS1, LATS2, YAP1, TAZ, AREG, CTGF, CYR61 and MYC) expression in METTL3-knockdown, CM-treated cells. (D-G) Detected effects of METTL3 and CM on N6-methyladenosine levels of Hippo signalling pathway-related genes in HMEC-1 cells. **P<0.01 and ***P<0.001. KEGG, Kyoto Encyclopedia of Genes and Genomes; CM, conditioned medium; YAP1, Yes-associated protein 1; NC, negative control; LV, lentivirus; NS, not significant; si-, small interfering.

To clarify the influence of METTL3 on signaling pathways in HMEC-1 cells after CM treatment, an RNA-sequencing analysis was carried out. Remarkably, the KEGG analysis showed that the Hippo pathway was significantly related to METTL3 (Fig. 3A). The Hippo pathway is an important signalling pathway that regulates cell proliferation (26). To identify changes within this pathway, RT-qPCR was used to detect the expression levels of key regulators and downstream targets. Our research results suggest that when HMEC-1 cells were treated with CM, interference with METTL3 significantly inhibited YAP1 and its downstream targets (Fig. 3B). When METTL3 was overexpressed, it significantly increased the expression level of YAP1 and its downstream targets (Fig. 3C). The results of MeRIP-qPCR demonstrated that the presence of METTL3 was directly associated with an increase in the m6A level of YAP1 in HMEC-1 cells (Fig. 3D). Moreover, it was also found that the m6A level of YAP1 was increased in CM-treated HMEC-1 cells (Fig. 3E). After interfering with METTL3 expression in HMEC-1 cells, the m6A level of YAP1 in HMEC-1 cells decreased (Fig. 3F). Additionally, interfering with METTL3 expression reduced the increase in the m6A level in HMEC-1 cells induced by CM treatment (Fig. 3G). These results indicate that YAP1 is the target gene of METTL3 in HMEC-1 cells with CM treatment.

### METTL3 promotes the nuclear translocation of the YAP1 protein

**Figure 4.**
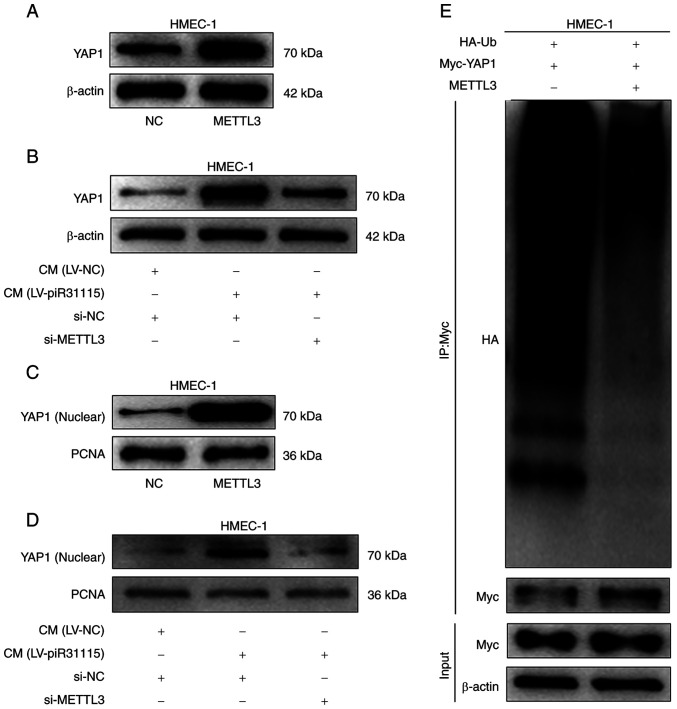
METTL3 regulates YAP1 gene expression in HMEC-1 cells. (A) WB detected YAP1 in METTL3-overexpressing cells. (B) WB was used to analyse YAP1 protein levels after CM treatment and METTL3 knockdown. (C) WB was utilized to assess YAP1 nuclear protein level in METTL3-overexpressing cells. (D) YAP1 nuclear protein levels after CM treatment and METTL3 knockdown were assessed by WB. (E) Ubiquitinated YAP1 level in METTL3-overexpressing cells was examined using WB. Data from three independent experiments; YAP1, Yes-associated protein 1; WB, western blotting; CM, conditioned medium; NC, negative control; LV, lentivirus; si-, small interfering; PCNA, proliferating cell nuclear antigen.

The regulatory effect of METTL3 on the YAP1 protein level was examined. WB results demonstrated that overexpression of METTL3 significantly increased the YAP1 expression level in HMEC-1 cells (Fig. 4A). Meanwhile, CM also enhanced the YAP1 protein expression level. However, interference with METTL3 suppressed YAP1 expression level when the cells were treated with CM (Fig. 4B). Nuclear protein was extracted from HMEC-1 cells with overexpression of METTL3 or treated with CM and it was found that YAP1 expression increased. Nevertheless, interfering with METTL3 could inhibit the promoting effect of CM on the expression of YAP1 nuclear protein (Fig. 4C and D). The detection of YAP1 ubiquitination in HMEC-1 cells revealed that the level of ubiquitinated YAP1 protein decreased after overexpression of METTL3 (Fig. 4E). The aforementioned results indicated that the upregulated expression of METTL3 in HMEC-1 cells promotes nuclear translocation of the YAP1 protein under CM treatment.

### METTL3 affects the proliferation and migration of HEMC-1 cells by regulating YAP1

**Figure 5.**
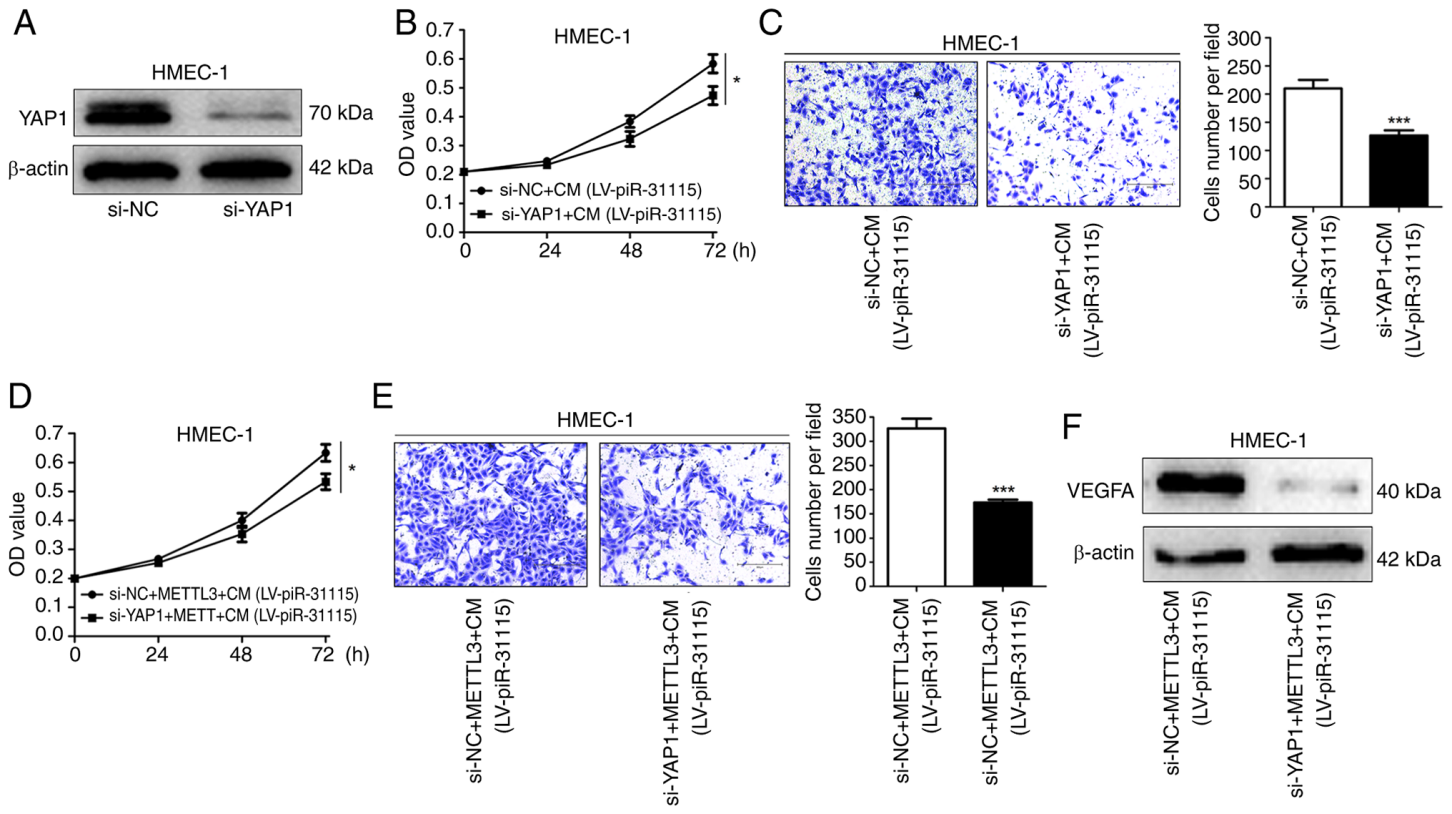
YAP1 promotes HMEC-1 cell proliferation and migration. (A) WB was used to determine YAP1 expression in YAP1-knockdown cells. (B) CCK-8 assay was used to assess proliferation after CM treatment and YAP1 knockdown. (C) Migration after CM treatment and YAP1 knockdown was evaluated by Transwell assays (magnification, ×100). (D) Cell proliferation after CM treatment, METTL3 overexpression or YAP1 knockdown was detected using CCK-8 assay. (E) Transwell assays were utilized to measure migration after CM treatment, METTL3 overexpression or YAP1 knockdown (magnification, ×100). (F) VEGFA expression after CM treatment, METTL3 overexpression or YAP1 knockdown was analysed by WB. Data are derived from three independent experiments. *P<0.05 and ***P<0.001. YAP1, Yes-associated protein 1; WB, western blotting; CCK-8, Cell Counting Kit-8; CM, conditioned medium; NC, negative control; LV, lentivirus; si-, small interfering.

When the expression of YAP1 in HMEC-1 cells was interfered with, the stimulatory effect of CM on cell proliferation and migration was significantly diminished (Fig. 5A-C). Moreover, it was also observed that when the expression of YAP1 in HMEC-1 cells was interfered with, the promoting influence of METTL3 on cell proliferation and migration was significantly weakened (Fig. 5D and E). Meanwhile, the expression level of VEGFA in HMEC-1 cells decreased (Fig. 5F). These results indicate that METTL3 affects the proliferation and migration of HEMC-1 cells by regulating YAP1.

### M6A-mediated regulation of YAP1 expression is identified by IGF2BP2 in HMEC-1 cells

**Figure 6.**
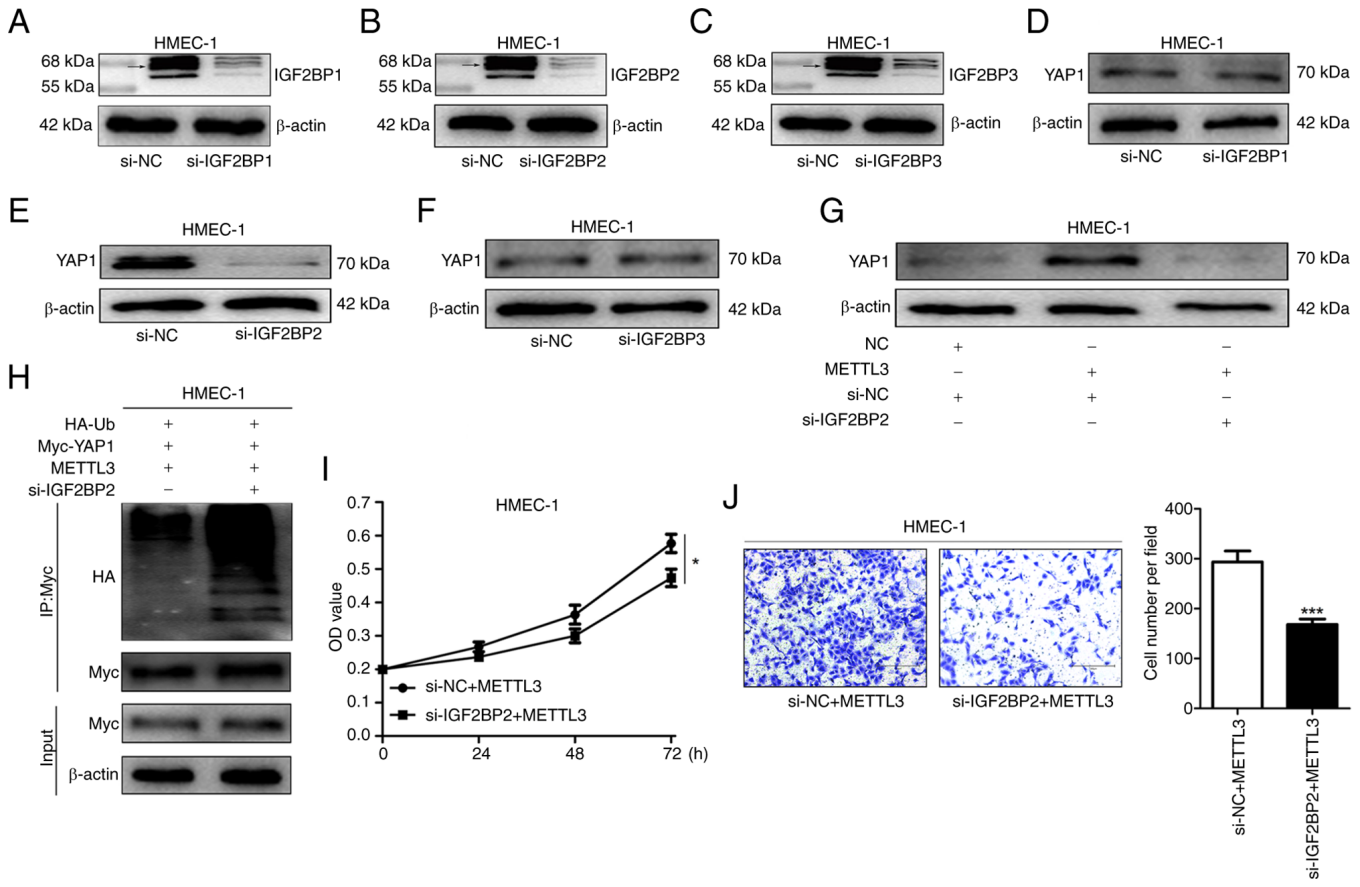
IGF2BP2 promotes YAP1 expression in HMEC-1 cells. (A-C) WB was used to detect IGF2BP1/2/3 expression in IGF2BP1/2/3-knockdown cells Due to the existence of multiple isoforms of IGF2BP1, IGF2BP2 and IGF2BP3 (possibly resulting from alternative splicing or post-translational modifications), multiple bands are presented in the WB. Among them, the bands around 65 kDa (as indicated by arrows) are the ones that were mainly considered to be the primary candidate bands representing the IGF2BP1, IGF2BP2 and IGF2BP3 proteins, and the subsequent analysis is mainly based on these bands. (D-F) WB was utilized to analyse IGF2BP1/2/3 expression in YAP1-expressing cells. (G) YAP1 expression in METTL3-overexpressing or IGF2BP2-knockdown cells was examined by WB. (H) Ubiquitinated YAP1 level in METTL3-overexpressing or IGF2BP2-knockdown cells was determined using WB. (I) Cell Counting Kit-8 assay was used to assess proliferation of METTL3-overexpressing or IGF2BP2-knockdown cells. (J) Transwell assays were used to evaluate migration of METTL3-overexpressing or IGF2BP2-knockdown cells (magnification, ×100). Data are derived from three independent experiments. *P<0.05 and ***P<0.001. YAP1, Yes-associated protein 1; WB, western blotting; si-, small interfering; NC, negative control.

Previous investigations have identified the IGF2BP family as m6A readers. This family comprises IGF2BP1, IGF2BP2 and IGF2BP3 (27). In order to elucidate the role of the IGF2BP family within the present study, siRNAs were employed to interfere with the expression of IGF2BP1, IGF2BP2 and IGF2BP3 in HMEC-1 cells (Fig. 6A-C). It is evident that only in the HMEC-1 cells with IGF2BP2 knockdown, the YAP1 protein expression level was markedly diminished (6D-F). Interestingly, when IGF2BP2 expression was simultaneously interfered with and METTL3 was overexpressed, the elevation in YAP1 expression level was suppressed, indicating that IGF2BP2 plays a crucial role in the process of METTL3 promoting YAP1 expression (Fig. 6G). Additionally, through co-IP experiments, it was discovered that the ubiquitination level of YAP1 in the si-IGF2BP2 group was increased compared with that in the si-NC group, suggesting that IGF2BP2 may maintain the stability of YAP1 by reducing its ubiquitination (Fig. 6H). Further investigations into the effects of IGF2BP2 on the proliferation and migration of HMEC-1 cells revealed that interfering with IGF2BP2 expression eliminated the promoting effect of METTL3 on the proliferation and migration of HMEC-1 cells, further confirming the pivotal role of IGF2BP2 in regulating YAP1-related cellular processes (Fig. 6I and J).

## Discussion

Cancer metastasis represents a formidable hurdle in the realm of cancer treatment, with angiogenesis, the formation of new blood vessels, assuming a pivotal role. In the context of TNBC, an especially aggressive subtype that lacks specific therapeutic targets, comprehending the mechanisms that underpin angiogenesis is of paramount significance. TNBC cells possess the propensity to detach from the primary tumour and metastasize to distant organs, and this process of theirs is highly dependent on the establishment of new blood vessels to sustain their growth and dissemination, as recently described (28,29). Angiogenesis not only furnishes the requisite nutrients and oxygen for the rapidly proliferating cancer cells but also functions as a conduit for their spread (30,31). By delving into the regulatory mechanisms of angiogenesis in TNBC, there is a chance of identifying novel therapeutic targets and devising more efficacious strategies to combat this lethal disease. Recent studies have found that ncRNAs, as tissue-specific molecules, play both oncogenic and tumour-suppressive roles in cancer progression, including cancer cell proliferation, metastasis, chemoresistance and stemness (14,32). In the present study, our focus lies on the role of piR-31115 within the TNBC cell-induced angiogenesis process and its associated molecular mechanisms.

piR-31115, being a constituent of the ncRNA family, is a member of the piRNA family (21). Du *et al* (20) have established that the expression of piRNA-31115 is abnormally upregulated in renal clear cell carcinoma tissues and have illustrated that it functions as an oncogene, thereby facilitating the progression of renal clear cell carcinoma. In the present research centered around TNBC, it was ascertained that the expression of piR-31115 was augmented in TNBC samples and TNBC cell lines. Concurrently, the outcomes of the cell co-culture experiment have disclosed an intriguing phenomenon: TNBC cells inherently possess the capacity to recruit HMEC-1 cells. Remarkably, when piR-31115 is overexpressed, this recruitment effect on HMEC-1 cells is conspicuously intensified. On the contrary, when the expression of piR-31115 is perturbed, the recruitment of HMEC-1 cells is diminished. Zhao *et al* (33) found through the research on the *in vitro* co-culture of MDA-MB-231 cells and human umbilical vein endothelial cells as well as the CD31 staining of tumour endothelial cells *in vivo* that piR-2158 has an inhibitory effect on the angiogenesis of breast cancer (33). Taken together, these results suggest that some piRNAs play a role in promoting angiogenesis in the context of TNBC.

Information exchange serves as the bedrock of interactions between cells. Cells are capable of secreting ‘signalling molecules’, such as exosomes, in a variety of ways (34). Upon other cells ingesting these ‘signalling molecules’, they proceed to regulate the relevant signalling pathways within the cell, prompting the cell to respond (35). In the present study, the expression of piR-31115 in HMEC-1 cells was significantly increased after being treated with CM. This finding suggests that HMEC-1 cells take up piR-31115, which is secreted by MDA-MB-231 cells. Currently, research on piRNAs in tumours is primarily focused on the regulatory mechanism of intracellular signaling pathways in cancer cells (17,18). However, there are scarce studies on the regulatory effects of cancer cell-derived piRNAs on other cells. In the present study, it was found that CM can increase the total m6A modification level and the expression level of METTL3 in HMEC-1 cells. These results suggest that piR-31115 derived from MDA-MB-231 cells may regulate HMEC-1 cells through m6A modification.

With the deepening of research in RNA epigenetics, RNA methylation has emerged as a pivotal factor in the intricate processes of tumorigenesis (36). Among them, m6A methylation, which refers to the methylation of the nitrogen atom N6 on the sixth carbon of adenosine (A) in the RNA molecule, is the most common form of mRNA modification (37,38). Research has demonstrated that it plays an important role in the progression of cancer (39–41). METTL3 is a key protein for m6A methylation in various types of cancers (42). However, there are few studies on METTL3 regulating tumour angiogenesis. Previous studies have shown that piRNAs need to bind to PIWI family proteins to exert their biological effects (43). The current results showed that piR-31115 derived from MDA-MB-231 cells could strengthen the binding of PIWIL4 to METTL3, thus increasing METTL3 expression level. Moreover, it was found that overexpression of METTL3 promotes the proliferation and migration of HMEC-1 cells. These results suggest that METTL3 plays an important role in promoting the angiogenesis.

The Hippo signalling pathway plays a significant role in organ development. Stimulated by extracellular growth inhibition signals, a cascade of kinases leads to the phosphorylation of the effector YAP1 and transcriptional coactivator TAZ, which remain in the cytoplasm and are degraded by ubiquitination to control cell proliferation and organ size (26). In the present study, it was found that interference with METTL3 expression in CM-treated HMEC-1 cells significantly affected the Hippo signalling pathway. Interestingly, only the m6A methylation level of the YAP1 gene was found to be regulated by METTL3. These findings confirmed that YAP1 was the target gene of METTL3 methylation. This is consistent with the study by Ni *et al* (44). The detection of YAP1 protein expression showed that METTL3 could prevent the ubiquitination-mediated degradation of YAP1 by altering its nuclear translocation, thereby enhancing the transcription of downstream target genes and leading to the proliferation and migration of HMEC-1 cells. m6A methylation-binding proteins often determine the fate of modified target genes. The IGF2BP protein family is a group of m6A reading proteins whose members enhance the stability of target genes (45). The present results showed that interference with IGF2BP2 expression results in an elevation of YAP1 ubiquitination levels, significantly inhibiting the proliferation and migration of HMEC-1 cells. This indicates that IGF2BP2 plays an important role in modulating the stability of YAP1 that undergoes m6A modification.

The findings of the present study strongly suggest that piR-31115 holds great potential for development as a drug target. From the perspective of drug research and development, multiple strategies can be explored to target piR-31115. For instance, small molecule inhibitors or antisense oligonucleotides that specifically bind to piR-31115 can be designed. By binding to piR-31115, they can prevent its interaction with PIWI family proteins or promote its degradation, thereby blocking the signalling pathway that promotes angiogenesis. Gene editing technologies such as the CRISPR-Cas system can also be used to directly regulate the expression of the piR-31115 gene, suppressing its abnormal function at the source. However, there are inevitably some challenges in the development process. For example, ensuring the high specificity of drug molecules for piR-31115 and avoiding affecting the functions of other normal RNAs. Meanwhile, it is necessary to construct an effective drug delivery system to increase the concentration and efficacy of drug molecules in tumour tissues and overcome problems such as difficulties in cell uptake and poor *in vivo* stability.

Our findings suggest that targeting the piR-31115/METTL3/YAP1/IGF2BP2 signalling pathway may offer a promising strategy for inhibiting tumour angiogenesis in TNBC. By interfering with this pathway, it is conceivable to suppress the proliferation and migration of vascular endothelial cells, thus impeding tumour growth and metastasis. This might potentially result in an augmentation of the PFS of patients with TNBC, which is of preponderant clinical importance. Future enquiries should focus on validating these findings *in vivo* and exploring the potential of devising novel therapies based on this mechanism. Moreover, the improvement in PFS could potentially translate into an enhanced quality of life for patients with TNBC, as it may defer the recurrence of the disease and the exigency for further aggressive treatments. Comprehending the role of this signalling pathway in angiogenesis not only affords insights into the pathophysiology of TNBC but also offers a glimmer of hope for more efficacious therapeutic interventions in the future.

However, it is of utmost importance to note that the current study is subject to certain limitations. Firstly, in our experiments, the MDA-MB-231 cell line was solely utilized. Whilst this cell line is widely employed in TNBC research, it is incapable of fully representing the heterogeneity of TNBC or other breast cancer subtypes. Different cell lines may display distinct genetic profiles, signalling pathways and responses to diverse factors. Consequently, the findings pertaining to the role of the piR-31115/METTL3/YAP1/IGF2BP2 signalling pathway in tumour angiogenesis may not be directly applicable to all TNBC cases or other breast cancer types. Future studies ought to incorporate a more extensive range of cell lines to augment the generalisability of the results. Secondly, the present study was conducted entirely *in vitro*. The *in vitro* environment lacks the complexity and dynamic interactions that take place *in vivo. In vivo*, tumours interact with surrounding stromal cells, immune cells and the extracellular matrix, which can exert a significant influence on angiogenesis and tumour progression. The absence of these factors in our *in vitro* model may have given rise to an incomplete comprehension of the physiological processes. As a result, *in vivo* studies are indispensable for validating and supplementing our *in vitro* findings and for providing a more comprehensive understanding of the role of this signalling pathway in TNBC angiogenesis.

**Figure 7.**
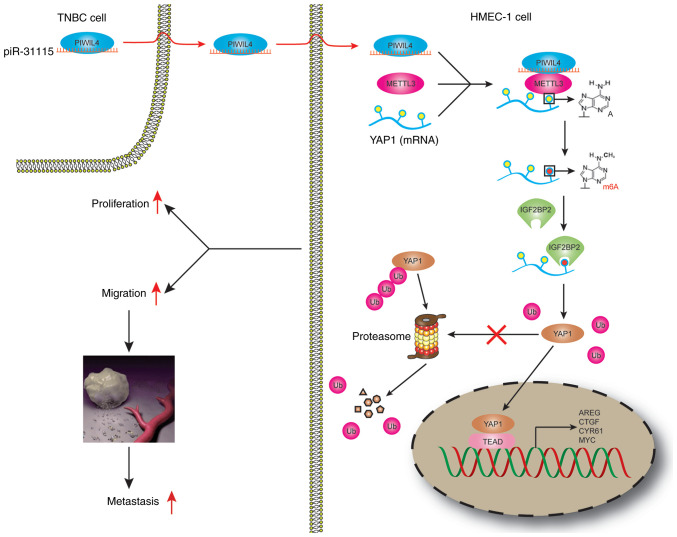
Schema illustrating the regulatory mechanism through which MDA-MB-231 cell-derived piR-31115 promotes the proliferation and migration of HMEC-1 cells. piR, Piwi-interacting RNA; TNBC, triple-negative breast cancer.

In conclusion, it was revealed that piR-31115 derived from MDA-MB-231 cells promotes HMEC-1 cells proliferation and migration. The internalization of piR-31115 into HMEC-1 cells augmented the m6A modification level of the METTL3-regulated gene YAP1, which is recognized by IGF2BP2. The increased expression of YAP1 ultimately led to alterations in the biological behaviour of HMEC-1 cells (Fig. 7).

## Figures and Tables

**Table I.** Sequences of primers used for reverse transcription-quantitative PCR.

Gene name	Primer sequence (5′-3′)
piR-31115	F: AGCCTGAGCAACATAGCGAG
	R: GTGCAGGGTCCGAGGTATTC
U6	F: CTCGCTTCGGCAGCACA
	R: AACGCTTCACGAATTTGCGT
METTL3	F: GAGTGTCGGAGGTGATT
	R: AGTACGGGTATGTTGAGC
METTL14	F: TGTACTTACAAGCCGATAT
	R: TAGCAGTGATGCCAGTT
WTAP	F: AGATGACCAACGAAGAAC
	R: CTAGTCGCATTACAAGGAT
ALKBH5	F: TTTTCCCCCTTTAGTCTCC
	R: CCCTTCACCAACTCCCAT
FTO	F: GGTGTCCCAAGAAATCGT
	R: CTGGTGGCAGGAAAGAGT
MST1	F: CACCGATTTACGCCAGAAAA
	R: GAAGTTCTCCTCCAGTTGTG
MST2	F: ACCTCTGGATTGTTATGGAGTACTG
	R: TCTGTGTATTTTTCTCATAAAGTGC
LATS1	F: CCACCCTACCCAAAACATCT
	R: TCACTCTCATCTTCCTTGGG
LATS2	F: GTGGACCTGTATGAATTGGG
	R: TGGTGGCTGTTGAAGGAGTT
YAP1	F: GCTACAGTGTCCCTCGAACC
	R: TCCTTCCAGTGTTCCAAGGT
TAZ	F: GAGCATAGAAGGCAGGTGAGCAAC
	R: GGCAAGGGCGGTGGGTAGG
AREG	F: CGGTCTCCACTCGCTCTTCC
	R: GGGCTCTCATTGGTCCTTCGC
CTGF	F: GCGAGGAGTGGGTGTGTGACG
	R: TGGACCAGGCAGTTGGCTCTAATC
CYR61	F: TGAGGTGCGGCCTTGTGGA
	R: CACTCAAACATCCAGCGTAAGTAA

F, forward; R, reverse; YAP1, Yes-associated protein 1; piR, Piwi-interacting RNA.

**Table II.** Sequences of si-METTL3, si-YAP1, si-IGF2BP1, si-IGF2BP2, si-IGF2BP3 and si-NC.

Name	Sequence (5′-3′)
si-METTL3	Sense: AGCUACAGAUCCUGAGUUAGAGA (dT)(dT)
	Antisense: UCUCUAACUCAGGAUCUGUAGCU (dT)(dT)
si-YAP1	Sense: GAGAUACUUCUUAAAUCACAUCG (dT)(dT)
	Antisense: CGAUGUGAUUUAAGAAGUAUCUC (dT)(dT)
si-IGF2BP1	Sense: CACCAUGAACAAGCUUUACAUCG (dT)(dT)
	Antisense: CGAUGUAAAGCUUGUUCAUGGUG (dT)(dT)
si-IGF2BP2	Sense: UGGAAUUGCAUGGGAAAAUCAUG (dT)(dT)
	Antisense: CAUGAUUUUCCCAUGCAAUUCCA (dT)(dT)
si-IGF2BP3	Sense: CACAAUGAACAAACUGUAUAUCG (dT)(dT)
	Antisense: CGAUAUACAGUUUGUUCAUUGUG (dT)(dT)
si-NC	Sense: UUCUCCGAACGUGUCACGU (dT)(dT)
	Antisense: ACGUGACACGUUCGGAGAA (dT)(dT)

si-, small interfering; NC, negative control; YAP1, Yes-associated protein 1.

## Data Availability

The data generated in the present study can be requested from the corresponding author.
